# Carbon Xerogels Hydrothermally Doped with Bimetal Oxides for Oxygen Reduction Reaction

**DOI:** 10.3390/ma12152446

**Published:** 2019-07-31

**Authors:** Abdalla Abdelwahab, Francisco Carrasco-Marín, Agustín F. Pérez-Cadenas

**Affiliations:** 1 Materials Science and Nanotechnology Department, Faculty of Postgraduate Studies for Advanced Sciences, Beni-Suef University, Beni-Suef 62511, Egypt; 2 Carbon Materials Research Group, Department of Inorganic Chemistry, Faculty of Sciences, University of Granada, Campus Fuentenueva s/n, ES18071 Granada, Spain

**Keywords:** carbon gels, mesoporosity, electrocatalysis, oxygen reduction reaction

## Abstract

A total of two carbon xerogels doped with cobalt and nickel were prepared by the sol–gel method. The obtained carbon xerogels underwent further surface modification with three binary metal oxides namely: nickel cobaltite, nickel ferrite, and cobalt ferrite through the hydrothermal method. The mesopore volumes of these materials ranged between 0.24 and 0.40 cm^3^/g. Moreover, there was a morphology transformation for the carbon xerogels doped with nickel cobaltite, which is in the form of nano-needles after the hydrothermal process. Whereas the carbon xerogels doped with nickel ferrite and cobalt ferrite maintained the normal carbon xerogel structure after the hydrothermal process. The prepared materials were tested as electrocatalysts for oxygen reduction reaction using 0.1 M KOH. Among the prepared carbon xerogels cobalt-doped carbon xerogel had better electrocatalytic performance than the nickel-doped ones. Moreover, the carbon xerogels doped with nickel cobaltite showed excellent activity for oxygen reduction reaction due to mesoporosity development. NiCo_2_O_4_/Co-CX showed to be the best electrocatalyst of all the prepared electrocatalysts for oxygen reduction reaction application, exhibiting the highest electrocatalytic activity, lowest onset potential E_onset_ of −0.06 V, and the lowest equivalent series resistance (ESR) of 2.74 Ω.

## 1. Introduction

The energy problem is one of the most important challenges the world is facing right now. Finding new sources of energy production and how to store this energy has become a major challenge. Lithium ion batteries are a kind of batteries that are used in portable electronics and electric vehicles [1]. Although lithium ion battery produces electricity with high energy density and low self-discharge, it presents some hazards as it contains a flammable electrolyte.

Fuel cells are electrochemical devices that are able to convert chemical energy into electrical energy when fuel and oxidant are supplied [2]. According to their working mechanism, fuel cell bears similarities in both batteries and engines, however, it has superior advantages as it does not need recharging and generates drinking water when the used fuel is hydrogen, so, it is considered as a “zero emission engine.” Because it is environmentally friendly, fuel cells find commercial application in transportation, stationary power generation, and in low power portable devices. Fuel cells are facing some difficulties that delay its entry into the market. These difficulties can be attributed to economic factors, materials designing problem, and inadequacies in electrochemical devices operation [2,3].

The fuel cell is a galvanic cell that consists of two electrodes, anode and cathode. The anodic fuel cell reaction is either the direct oxidation of hydrogen or the oxidation of methanol. The cathodic fuel cell reaction is usually oxygen reduction reaction (ORR) and in most cases the source of oxygen is air. The major factor that limits the fuel cell performance is the cathodic oxygen reduction reaction (ORR), as it consists of several steps in which molecular oxygen dissociates at the catalyst surface and combines with hydrogen ions [4,5]. Different factors can influence the reaction kinetics at the electrode surface, but still the electrocatalyst itself has the major effect. There are two pathways for the oxygen reduction reaction in aqueous electrolyte: four-electron and two-electron pathways. The direct four-electron pathway is preferable because the Faradaic efficiency of the reaction is greater; also it does not involve peroxide species in the solution.

To date, Pt and its alloys are the best known electrocatalysts for the ORR [6,7,8,9]. In order to reduce the cost of using pure Pt metal as a catalyst, alloying Pt with another metal reduces the cost; however the metal leaches away gradually [9,10], resulting in the loss of performance that reduces the total fuel cell efficiency and limits their market use. The current fuel cell technology is based on the development of non-precious metals and Pt-free electrocatalysts [10]. Transition metal oxides are new materials that exhibit excellent activity in many applications because of their rich redox reactions, higher conductivity than simple oxides, and availability of active sites. Wang et al. [11] fabricated graphene-nickel cobaltite nanocomposite (GNCC) that was used as a positive electrode in supercapacitors application. Higher capacitance was obtained for GNCC (618 F·g^−1^) compared with graphene-Co_3_O_4_ (340 F·g^−1^) or graphene-NiO (375 F·g^−1^) due to the rich numbers of Faradaic reactions on the nickel cobaltite. Moreover, Genqiang Zhang et al. [12] synthesized NiCo_2_O_4_-rGO hybrid nanosheets electrocatalysts for the oxygen reduction reaction. He found a comparable current density and onset potential with those of commercial Pt/C catalysts. In another study [13], cobalt ferrite thin films were prepared and tested as anode for lithium-ion batteries.

Carbon nanomaterials such as carbon nanotubes and graphene were applied as electrocatalysts for ORR application [14,15] and exhibited good performance due to their surface active sites that are necessary for reactants adsorption, bond-breaking and new bond-formation, and products desorption. A new emerging class of carbon nanomaterials is carbon gel [16,17], which has good electrochemical properties and high surface areas. Recently, carbon gels were tested as electrodes in methanol oxidation [18], supercapacitors [19], environmental applications [20,21], and oxygen reduction reaction (ORR) [22,23]. Carbon gels, in both of its forms xerogels and aerogels, offer the opportunity to be used as it is or doped with metals which enhances its electrochemical activity as the metal doping influences the obtained surface area and morphology [24,25,26].

To the best of our knowledge, bimetal oxides-doped carbon xerogels have not been tested before as electrocatalysts for oxygen reduction reaction in basic medium. So, in this work carbon xerogels were prepared by sol–gel process using two metal salts as polymerization catalysts namely cobalt acetate and nickel acetate to investigate the role of the metal catalyst. Moreover, the resultant two carbon xerogels were further doped with different binary metal oxides of nickel cobaltite, nickel ferrite, and cobalt ferrite through the hydrothermal method. The prepared samples were employed as electrocatalysts for oxygen reduction reaction, as this is one of our main goals to study the effect of bimetal oxides on the morphology of carbon gels and its activity toward ORR.

## 2. Materials and Methods

### 2.1. Preparation of Carbon Xerogel

The used monomers for preparation of carbon xerogels were resorcinol (R) and formaldehyde (F) with a molar ratio of R/F = 1/2. These monomers were dissolved in water (W) in the presence of cobalt acetate and nickel acetate as the polymerization catalysts. The amount of cobalt and nickel in the final carbon structure was calculated to be 6 wt.% and the used molar ratio between resorcinol and water R/W was 1/17. After stirring, the clear solution was filled in glass molds and placed in the oven for one day at 40 °C then five days at 80 °C. The obtained organic gel was placed in acetone for 3 days to allow solvent exchange to save the porosity during the drying method. The organic gels were dried using the microwave drying method (domestic Samsung microwave F600G, Samsung, MWF600G, Beijing, China) under Ar-gas flow at power of 10% for 10 min) to get their corresponding organic xerogels, followed by the carbonization process in tube furnace (Carbolite Gero single zone EVA, Carbolite Gero Neuhausen, Germany) at 900 °C for 2 h with a heating rate of 1 °C/min to get the carbon xerogels.

### 2.2. Binary Metal Oxides Surface Modification (XY_2_O_4_/CX)

The obtained cobalt and nickel-doped carbon xerogels were further doped with three different bimetal oxides through the hydrothermal process. Nickel cobaltite (NiCo_2_O_4_), nickel ferrite (NiFe_2_O_4_), cobalt ferrite (CoFe_2_O_4_) were chosen to be used as the bimetal oxides for doping.

In the synthesis of NiCo_2_O_4_/Co-CX and NiCo_2_O_4_/Ni-CX, typically 120 mg of carbon xerogel was dispersed into 40 mL *N*,*N*-dimethylformamide (DMF). Then, Ni(Ac)_2_·6H_2_O (0.125 gm; 0.5 mmol), Co(Ac)_2_·4H_2_O (0.250 gm; 1 mmol), and urea (0.360 gm; 6 mmol) were dissolved in 30 mL solution of H_2_O and ethylene glycol with a volumetric ratio of 1:2. The two solutions were sonicated for 15 min and were placed in a polytetrafluoroethylene lined stainless steel autoclave at 180 °C for 12 h. The black precipitate obtained after the hydrothermal reaction was collected by centrifugation (4000 rpm for 5 min), washed several times with water and ethanol, dried at 60 °C for 12 h, and finally calcined at 360 °C for 3 h with a heating rate of 5 °C/min.

The same procedure was followed for the preparation of nickel ferrite (NiFe_2_O_4_) and cobalt ferrite (CoFe_2_O_4_) doped carbon xerogel except with replacing the metal salts with the appropriate ones and maintaining the molar ratios between the two salts at 1:2.

### 2.3. Characterization

The prepared samples were fully characterized with different characterization techniques. Sample surface area and porosity were characterized using surface area analysis with N_2_ adsorption at −196 °C using the Quantachrome instrument (Quadrasorb, Boynton Beach, FL, USA) and then by applying the Brunauer-Emmett-Teller (BET) equation the isotherms were obtained. Before porosity analysis, the samples were outgassed for 12 h at 110 °C under high vacuum of 10^−6^ mbar. The mesoporosity of the prepared materials were calculated by applying the density functional theory (DFT) equation to the adsorption part of N_2_-isotherms. Moreover, the samples morphology and particle size distribution were analyzed using scanning electron microscopy (SEM) and high resolution transmission electron microscopy (HRTEM), respectively. SEM analysis was performed using Zeiss SUPRA40VP instrument (Carl Zeiss AG, Oberkochen, Germany), equipped with both SE and BSE detectors and X-Max 50 mm energy dispersive X-ray microanalysis system.

HRTEM was carried out with FEI Titan G2 60–300 microscope (FEI, Eindhoven, Netherlands) with a high brightness electron gun (X-FEG) operated at 300 KV and equipped with a Cs image corrector (CEOS) and analytical electron microscopy (AEM) with a SUPER-X silicon-drift window-less EDX detector.

XRD analysis was carried out with a BRUKER D8 DISCOVER diffractometer (BRUKER, Rivas-Vaciamadrid, Spain) equipped with a IµS Cu microsource, operating at 50 KV, 1 mA, and 50 W at 25 °C, using a Cu Kα (λ = 15,406 Å) radiation, a Multilayer Optics Monochromator (Quazar Optics: Montel type 2-dim beam shaping) (Incoatec, Geesthacht, Germany), and a PILATUS3R 100K-A detector (Dectris Ltd, Baden, Switzerland). Diffraction patterns were recorded between 10° and 80° (2θ) with a step of 0.02° and a time per step of 40 s. The average crystal size was determined using the Scherrer equation.

X-ray photoelectron spectroscopy (XPS) measurements were carried out with a physical electronics ESCA 5701 (PHI, Chanhassen, MN, USA) operating at 12 KV and 10 mA and equipped with a MgK X-ray source (*hν* = 1253.6 eV). The obtained binding energy values are referred to C1s, O1s, and N1s peaks at 284.6, 529.3, and 399.3, respectively.

### 2.4. Electrode Preparation for ORR

A total of 5 mg of the prepared carbon material was dispersed into 400 µL of isopropanol and 30 µL of nafion solution (5 wt.%), and then sonicated (sonication bath, Samarth electronics, for 15 min). Ten microliter of the suspended solution was deposited into a glassy carbon electrode with a diameter of 3 mm and dried under an infrared lamp for 5 min (100 W, R95, Philips, Madrid, Spain).

### 2.5. Electrochemical Measurements

The electrochemical measurements were carried out using a biologic multichannel VMP3 potentiostat (BioLogic, Seyssinet-Pariset, France). A three-electrode electrochemical cell was used during the analysis of electrodes performance in which Ag/AgCl and Pt electrodes were used as reference and counter electrodes, respectively. The used electrolyte was 0.1 M potassium hydroxide (KOH), which is first saturated with nitrogen then saturated with O_2_ to evaluate the electrocatalyst performance in the absence and presence of oxygen. Different electrochemical techniques were used in the electrode evaluation: (i) cyclic voltammetry (CV), (ii) linear sweep voltammetry (LSV), and (iii) electrochemical impedance spectroscopy (EIS).

The cyclic voltammetry and linear sweep voltammetry were carried out in a potential range between 0.4 to −0.8 V. Two scan rates of 5 mV·s^−1^ and 50 mV·s^−1^ were used with CV and different rotation speeds from 500 to 4000 rpm were employed for LSV at 5 mV·s^−1^ in order to be able to apply the Koutecky–Levich model for evaluating the electrocatalyst performance and calculating the number of transferred electrons.

(1) $$\frac{1}{j}=\frac{1}{j_{k}}+\frac{1}{B\omega^{0.5}}$$

(2) $$B=0.2nF{\left(D_{O_{2}}\right)}^{2/3}v^{-1/6}C_{O_{2}}$$

where *j*, current density; *j_k_*, kinetic current density; *ω*, rotation speed; *F*, Faraday constant; *D_O_*_2_, oxygen diffusion coefficient (1.9·10^−5^cm^2^·s^−1^); *ν*, viscosity (0.01cm^2^·s^−1^); *C_O_*_2_, oxygen concentration (1.2·10^−6^ mol·cm^−3^).

## 3. Results

Table 1 is constructed by applying the BET equation for the obtained isotherms of N_2_ adsorption at 77 K. The BET surface areas (S_BET_) ranged between 50 to 156 m^2^.g^−1^, as can be seen in Table 1. The highest surface areas were obtained for the samples doped with nickel ferrite NiFe_2_O_4_, which means these samples have high microporosity as appears from their pore size (Lo) data.

**Figure 1:**
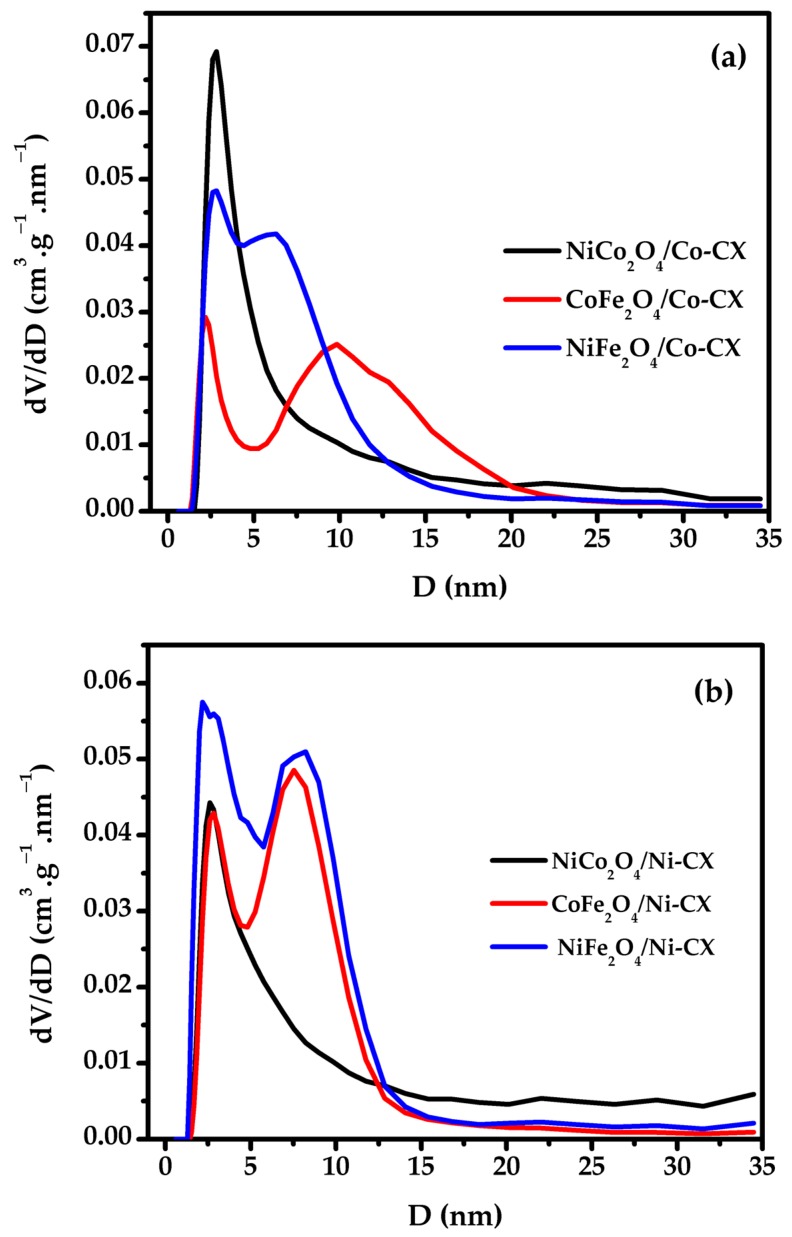
DFT analysis for adsorption part of N_2_-adsorption/desorption isotherms, (**a**) bimetal oxides doped cobalt xerogels, (**b**) bimetal oxides doped nickel xerogels.

Doping of carbon xerogel with bimetal oxides promote mesoporosity of carbon structure and this mesoporosity is confirmed by applying the DFT equation to the adsorption part for the obtained N_2_- isotherms (Figure 1). The DFT pores diameters (L_o_(DFT)) ranged from 2.18–2.84 nm and the samples doped with nickel cobaltite, NiCo_2_O_4_, showed homogenous particle size distribution. The mesoporous character of these samples is confirmed by the type-IV shape of their corresponding adsorption–desorption isotherms (Figure S1 in Supplementary Materials), also showing all of their significant hysteresis cycles.

**Figure 2:**
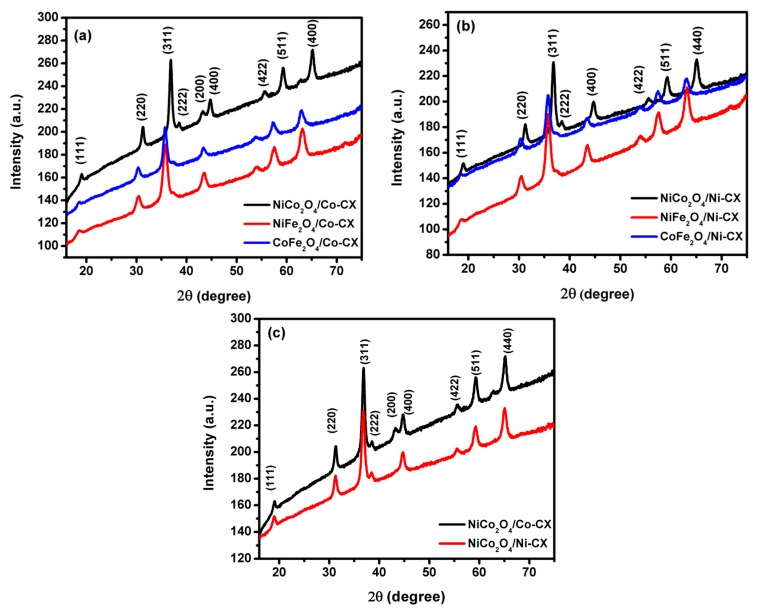
The XRD patterns for (**a**) bimetal oxides doped cobalt xerogels, (**b**) bimetal oxides doped nickel xerogels and (**c**) nickel cobaltite doped cobalt and nickel carbon xerogels.

The existence of the binary metal oxides inside the carbon matrix is confirmed by XRD analysis (Figure 2) in which the crystallinity of the bimetal oxides is confirmed by the XRD pattern with the absence of any contaminated peaks. The XRD pattern of nickel cobaltite doped carbon xerogels and their corresponding planes is presented in Figure 2c.

**Figure 3:**
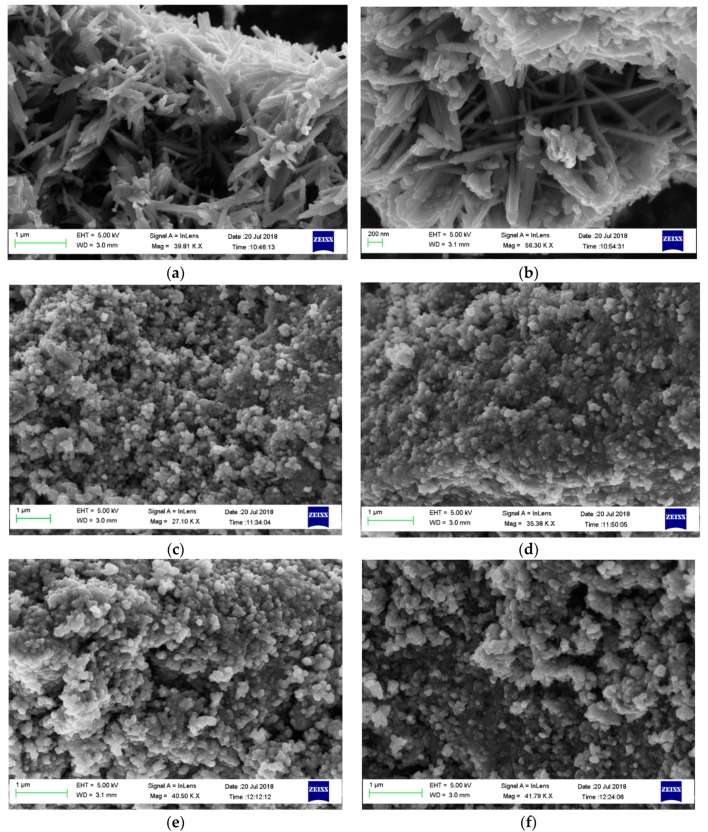
Scanning electron microscopy (SEM) images for (**a**) NiCo_2_O_4_/Co-CX, (**b**) NiCo_2_O_4_/Ni-CX, (**c**) NiFe_2_O_4_/Co-CX, (**d**) NiFe_2_O_4_/Ni-CX, (**e**) CoFe_2_O_4_/Co-CX, and (**f**) CoFe_2_O_4_/Ni-CX.

The morphology of the prepared samples is revealed from the SEM images (Figure 3). The carbon xerogel undergoes morphology transformation from continued connected spherical particles to nano-needle like structure when doped with nickel cobaltite (Figure 3a,b), while it maintains its original morphology when doped with nickel ferrite (Figure 3c,d) or cobalt ferrite (Figure 3e,f).

**Figure 4:**
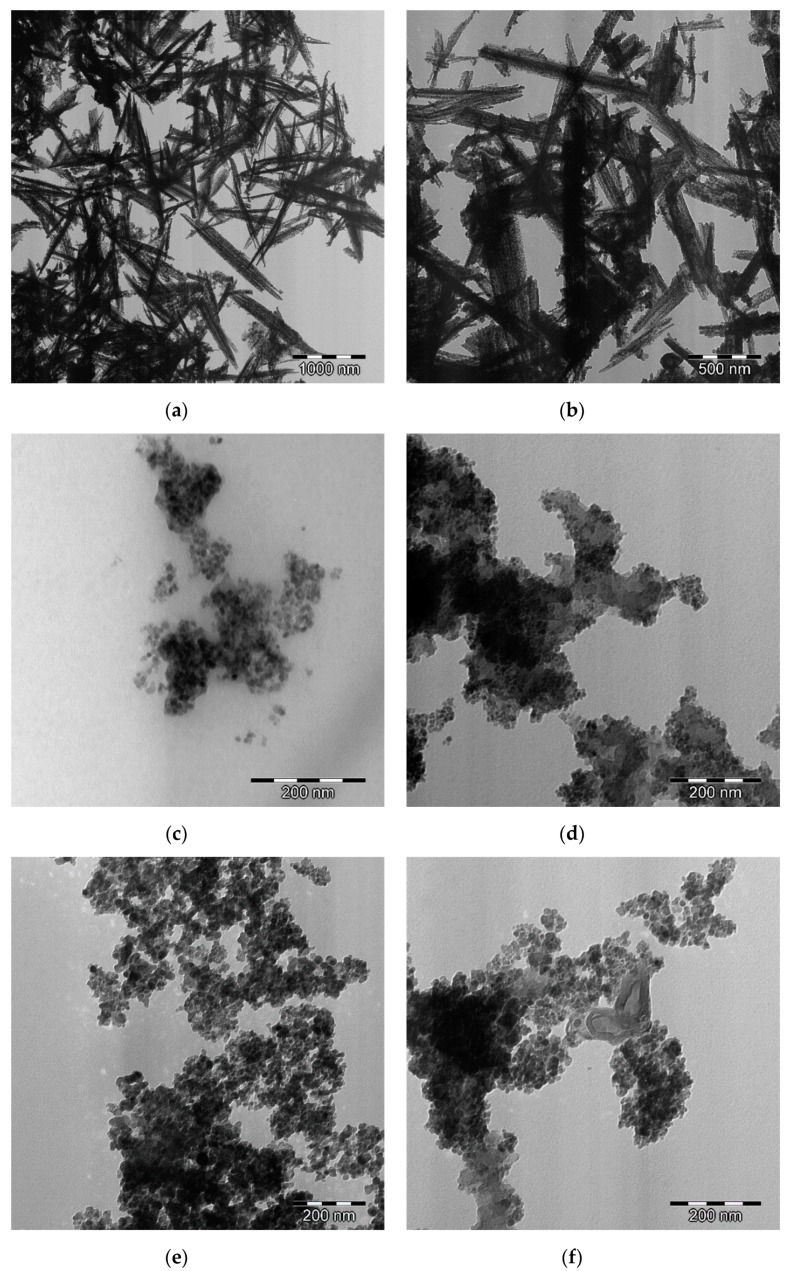
High resolution transmission electron microscopy (HRTEM) images for (**a**) NiCo_2_O_4_/Co-CX, (**b**) NiCo_2_O_4_/Ni-CX, (**c**) NiFe_2_O_4_/Co-CX, (**d**) NiFe_2_O_4_/Ni-CX, (**e**) CoFe_2_O_4_/Co-CX, and (**f**) CoFe_2_O_4_/Ni-CX.

The homogeneity and dispersity of the doped bimetal oxides nanoparticles inside the carbon matrix is studied from the TEM images (Figure 4). The metal nanoparticles are well dispersed inside the carbon structure in case of the doped samples with nickel ferrite (Figure 4c,d) and cobalt ferrite (Figure 4e,f). In addition, the nano-needle structure for nickel cobaltite with different lengths and widths was formed in both cobalt-doped carbon xerogels (Co-CX, Figure 4a) and nickel-doped carbon xerogels (Ni-CX, Figure 4b) [18].

**Figure 5:**
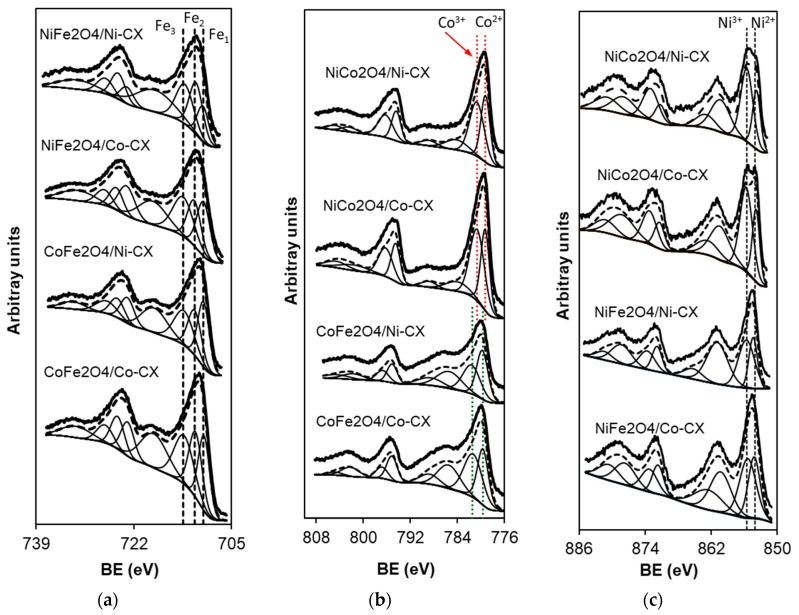
Deconvolution of the XP spectra for the prepared materials. (**a**) NiFe_2_O_4_ and CoFe_2_O_4_ doped carbon xerogels, (**b**) NiCo_2_O_4_ and CoFe_2_O_4_ doped carbon xerogels and (**c**) NiCo_2_O_4_ and NiFe_2_O_4_ doped carbon xerogels.

**Figure 6:**
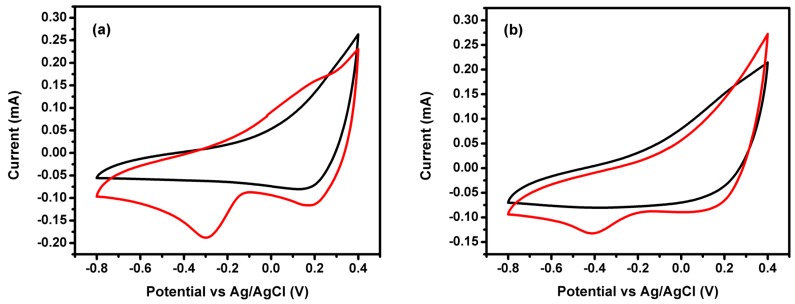
Cyclic voltammograms (CV) of (**a**) NiCo_2_O_4_/Co-CX in both nitrogen (black) and oxygen (red) saturated electrolyte and (**b**) NiCo_2_O_4_/Ni-CX in both nitrogen (black) and oxygen (red) saturated electrolyte.

Figure 5 shows the XP spectra of the prepared electrocatalysts in the presence of the metal cations in divalent and trivalent oxidation states. The prepared electrodes were tested as electrocatalysts for ORR application and their cyclic voltammograms for nickel cobaltite doped ones are presented in Figure 6.

**Figure 7:**
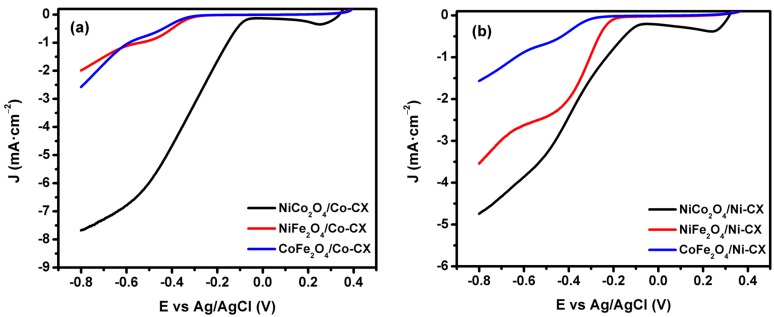
Linear sweep voltammograms (LSV) for (**a**) Co-CX and (**b**) Ni-CX doped with different bimetal oxides.

**Figure 8:**
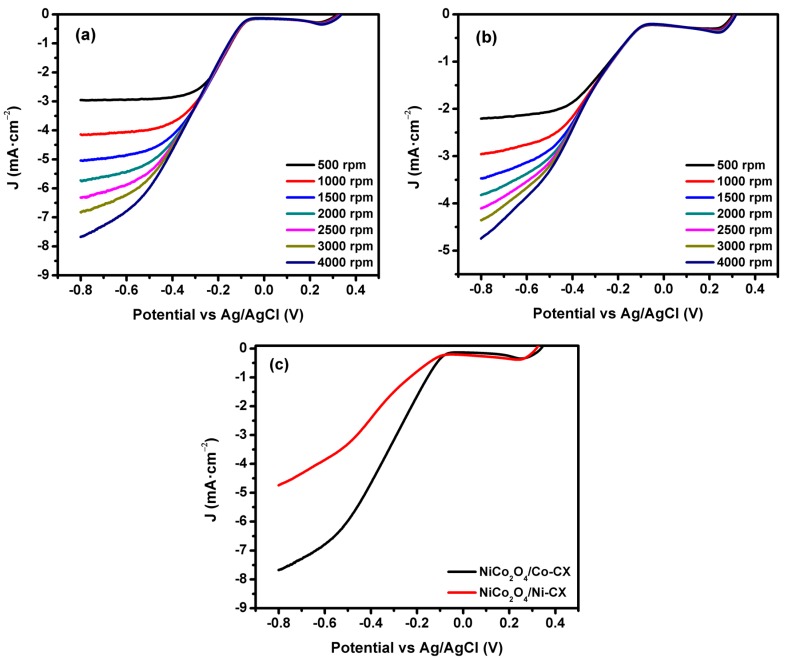
Linear sweep voltammograms (LSV) for (**a**) NiCo_2_O_4_/Co-CX, (**b**) NiCo_2_O_4_/Ni-CX at 5 mV.s^−1^ with different speeds from 500 rpm to 4000 rpm and (**c**) comparing the LSV for NiCo_2_O_4_/Co-CX and NiCo_2_O_4_/Ni-CX at 4000 rpm.

**Figure 9:**
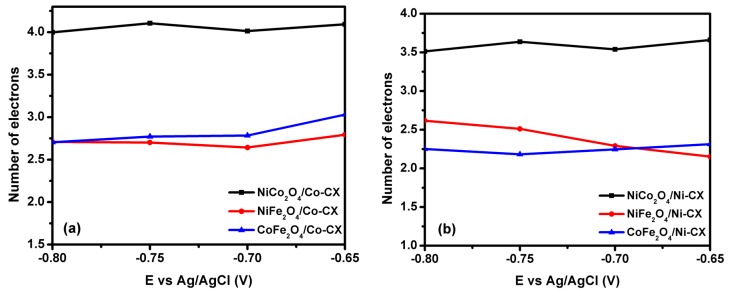
Variation of number of electron transferred with E vs. Ag/AgCl for bimetal oxides doped (**a**) Co-CX and (**b**) Ni-CX.

The linear sweep voltammetry (LSV) technique is used with rotating desk electrode (Figure 7), and the electrocatalytic activity at different rotation speed is shown in Figure 8. The data obtained from LSV were used in order to apply the Koutecky–Levich model for the determination of the number of electrons transferred (Figure 9).

**Figure 10:**
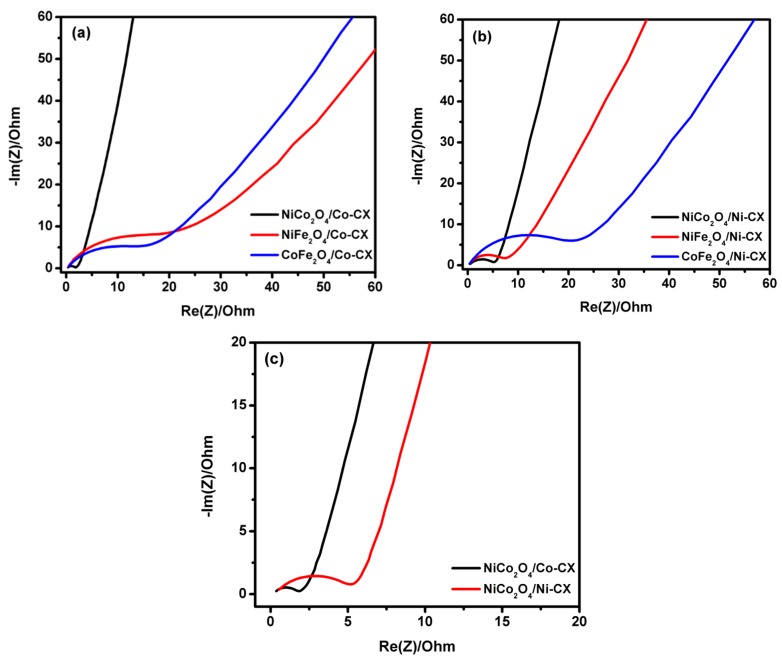
Nyquist plots obtained from EIS for bimetal oxides doped (**a**) Co-CX, (**b**) Ni-CX and (**c**) nickel cobaltite doped cobalt and nickel carbon xerogels.

Electrochemical impedance spectroscopy (EIS) is an important technique for the evaluation of the performance of an electrode in certain applications by calculating the electrode resistance and equivalent series resistance (ESR). The EIS was performed by applying a frequency range from 100 KHz to 1 mHz with a sinusoidal signal amplitude of 10 mV, and the data obtained from EIS is shown in Figure 10.

## 4. Discussion

Samples doped with nickel cobaltite have well developed mesoporosity and in case of NiCo_2_O_4_/Co-CX the mean pore size is 2.98 nm, while for NiCo_2_O_4_/Ni-CX is 2.20 nm. The mesoporosity development is an indication for better accessibility of electrolyte ions inside the carbon structure, which in turn make these materials good electrocatalysts in catalysis application [27,28,29].

Energy-dispersive X-ray spectroscopy (EDXS) analysis also confirmed the presence of the different metals in the samples. Figure S2 contains the analysis carried out on the sample NiCo_2_O_4_-CoCX, as an example.

Determination of mean particle sizes for the prepared samples was carried out by applying Scherrer equation for the obtained XRD patterns (Table 2). Higher particle sizes were obtained for the nickel cobaltite doped carbon xerogels. The higher mean particle sizes for nickel cobaltite doped samples indicates higher active sites in these samples which promote the electrocatalytic reduction of oxygen. For example, in NiCo_2_O_4_/Co-CX the mean particle size is about 25.5 nm while for NiFe_2_O_4_/Co-CX and CoFe_2_O_4_/Co-CX is 19.8 and 21.8 nm, respectively. Likewise for Ni-CX series the NiCo_2_O_4_/Ni-CX has the highest particle size of 24.1 nm.

Table 3 collects the binding energies (B.E.) and chemical composition corresponding to carbon, oxygen, and nitrogen with respect to the chemical composition analysed by XPS. The oxygen peak at lowest B.E. 529.8 ± 0.3 eV corresponds to the oxygen atoms bond to transition metal cations with oxidation states +2 and +3.

On the other hand, the XPS results corresponding to the region Fe2p are collected in Table 4 and Figure 5. Fe2p_3/2_ peaks centred at 709.9 ± 0.2, 711.2 ± 0.3, and 713.2 ± 0.2 eV have been assigned to Fe^2+^ situated in octahedral holes (Fe_1_), Fe^3+^ situated in octahedral holes (Fe_2_), and Fe^3+^ situated in tetrahedral holes (Fe_3_), respectively. This means that iron is forming part of compounds type $M_{x}^{2+}M_{1-x}^{3+}[{\mathrm{Fe}}_{y}^{2+}{\mathrm{Fe}}_{1-y}^{3+}]O_{4}$ [30] being M = Co and/or Ni.

Different Co2p_3/2_ peaks with respect to Co2p spectra peaks have been deconvoluted and assigned as the following: In the case of CoFe_2_O_4_ phases the peaks centred at 779.6 ± 0.1 correspond to Co^2+^ situated in tetrahedral holes whereas those centred at 781.5 ± 0.2 correspond to Co^2+^ situated in octahedral holes [30], therefore these metals deposited on the surface of the samples are forming part of the compounds type $({\mathrm{Co}}_{x}^{2+}{\mathrm{Fe}}_{y}^{3+})[{\mathrm{Fe}}_{z}^{2+}{\mathrm{Fe}}_{1-y}^{3+}{\mathrm{Co}}_{1-x}^{2+}]O_{4}$ where cations in parenthesis are situated in tetrahedral positions while cations in brackets are situated in octahedral positions. However, in the case of NiCo_2_O_4_ phases the peaks centred at 779.2 ± 0.1 correspond to Co^2+^ situated in octahedral holes whereas those centred at 780.6 ± 0.2 correspond to Co^3+^ situated in tetrahedral holes [4,31].

Finally, the XPS spectra of Ni2p region shows peaks at 854.2 ± 0.1 y 871.7 ± 0.2 eV which correspond to Ni^2+^ as well as peaks at 855.9 ± 0.3 y 873.5 ± 0.2 eV corresponding to Ni^3+^ cations [31]. Therefore, XPS results show the metals as divalent or trivalent species in all the cases. Taking into account all this XPS analysis, we can conclude that the different phases that have been synthetized and supported on the different samples of this work correspond with the stoichiometries collected in Table 5.

For ORR application, there is a reduction peak for oxygen saturated electrolyte for both NiCo_2_O_4_/Co-CX (Figure 6a) and NiCo_2_O_4_/Ni-CX (Figure 6b). This reduction peak is absent when the electrolyte is saturated with nitrogen (Black line), which means that these electrodes have electroactivity toward oxygen reduction reaction. Also, as it can be seen from the linear sweep voltammograms (LSV) (Figure 7) that the electrocatalytic activity for the nickel cobaltite doped carbon xerogels is higher than that for samples doped with nickel ferrite or cobalt ferrite and the onset potential for that sample is lower because of the increase in mesoporosity that allows higher accessibility for the electrolytic ions to access the pores (Table 1). The onset potentials E_onset_ for all samples are compiled in Table 6, in which the lowest onset potentials E_onset_ of −0.06 V is obtained for NiCo_2_O_4_/Co-CX. Similar data was obtained for NiCo_2_O_4_/Ni-CX with onset potential E_onset_ of −0.07 V. As it can be seen, samples doped with nickel ferrite and cobalt ferrite have comparable onset potentials.

In order to evaluate the number of transferred electrons during the reaction for each electrocatalyst, linear sweep voltammetry was carried out at 5 mV·s^−1^ at different rotating speeds from 500 rpm to 4000 rpm, in order to apply the Koutecky–Levich model (Figure 8). The LSV for NiCo_2_O_4_/Co-CX is presented in Figure 8a, in which the activity is promoted by increasing the rotating speed due to better diffusion of the electrolyte ions inside the pores. LSV data for NiCo_2_O_4_/Ni-CX are shown in Figure 8b. At the same rotating speed of 4000 rpm (Figure 8c), the activity of NiCo_2_O_4_/Co-CX to oxygen reduction is higher than that of NiCo_2_O_4_/Ni-CX, indicating their current densities.

The data obtained from fitting the linear sweep voltammograms to the Koutecky–Levich model (Figure 9) confirms that there is a promotion in the number of electrons transferred during the oxygen reduction reaction by doping the carbon xerogels with NiCo_2_O_4_. For example, in case of NiCo_2_O_4_/Co-CX (Figure 9a) the reaction takes place by the four electron pathway which is the favored one for oxygen reduction reaction (Table 6). While in case of NiCo_2_O_4_/Ni-CX and the rest of the electrocatalysts, the reaction occurs by both two and four electrons transfer pathways.

The electrochemical impedance spectroscopy (EIS), were performed for all prepared samples using the two electrode configuration in which 6 M KOH was used as the electrolyte in order to evaluate the electrode resistance and equivalent series resistance ESR (Figure 10). The activity of nickel cobaltite doped carbon xerogels toward ORR can also be attributed to the good electrical conductivity of nickel cobaltite relative to nickel ferrite or cobalt ferrite [32] as it can be seen from the Nyquist plots with lower electrode resistance for these samples (Figure 10). The equivalent series resistance ESR for the prepared samples was calculated from the Nyquist plot and is compiled in Table 6. Figure 10a shows the Nyquist plots for cobalt doped carbon xerogels (Co-CX) with the three bimetal oxides, the lowest ESR was obtained for NiCo_2_O_4_/Co-CX which equals to 2.74 Ω that reveals higher electrical conductivity and higher electrochemical performance to oxygen reduction. Likewise in case of nickel doped carbon xerogels (Ni-CX) (Figure 10b), the lowest ESR was achieved for NiCo_2_O_4_/Ni-CX with 6.18 Ω. Moreover, comparing Co-CX and Ni-CX doped NiCo_2_O_4_ (Figure 10c), the activity of carbon xerogels doped with cobalt is higher than that of nickel doped one because of the development of mesoporosity and lower electrode resistance, this tendency is in good agreement with our previous published work [23]. On the other hand, by comparing our electrocatalyst NiCo_2_O_4_/Co-CX with the ones in previously published materials such as NiCo_2_O_4_-rGO hybrid nanosheets in the same conditions [12], lower onset potential of −0.06 V was found compared to −0.073 V vs. Ag/AgCl indicating higher electrocatalytic activity to ORR. In addition, the ORR current density at a rotating speed of 2500 rpm and at −0.8 V vs. Ag/AgCl for NiCo_2_O_4_/Co-CX is about −6.3 mA.cm^−2^ while for NiCo_2_O_4_-rGO is about −2.0 mA·cm^−2^. The higher activity for ORR is also confirmed with the calculated number of electrons transferred which in case of NiCo_2_O_4_/Co-CX nanocomposite is n = 4.0 while for NiCo_2_O_4_-rGO hybrid nanosheets n = 3.8.

## 5. Conclusions

Binary metal oxides doped carbon xerogels were successfully prepared by the sol–gel process followed by a designed hydrothermal method. For all the prepared materials, the metal cations exist as divalent and trivalent species that occupy both the corresponding tetrahedral and octahedral positions in the crystal structure. The presence of metal cations inside the carbon xerogel structure develops the mesoporosity that makes these materials promising electrocatalysts for ORR. Nickel cobaltite doped carbon xerogels developed a new nano-needle like structure morphology and showed the highest electrocatalytic performance and lowest onset potential for oxygen reduction reaction. In general, development of mesoporosity of carbon xerogel together with increasing its electrical conductivity by bimetal oxides doping, especially nickel cobaltite based phase, improved the electrocatalytic performance in oxygen reduction reaction.

## Figures and Tables

**Table 1:** Surface area analysis.

Sample	S_BET_	W_0_(N_2_)	L_0_(N_2_)	V_0.95_(N_2_)	V_meso_(N_2_)	S_DFT_	V_DFT_	L_0_(DFT)
	m^2^/g	cm^3^/g	nm	cm^3^/g	cm^3^/g	m^2^/g	cm^3^/g	nm
NiFe_2_O_4_/Ni-CX	156	0.06	1.91	0.46	0.40	185	0.47	2.18
NiCo_2_O_4_/Ni-CX	57	0.02	2.20	0.27	0.24	103	0.31	2.60
CoFe_2_O_4_/Ni-CX	84	0.03	1.98	0.35	0.31	127	0.36	2.84
NiFe_2_O_4_/Co-CX	112	0.04	1.77	0.33	0.29	139	0.37	2.84
NiCo_2_O_4_/Co-CX	64	0.03	2.98	0.30	0.27	128	0.32	2.84
CoFe_2_O_4_/Co-CX	50	0.02	1.48	0.27	0.25	85	0.29	2.18

W_0_ is the micropore volume, L_0_ is the pore size, V_0.95_ is the pore volume at relative pressure of 0.95, V_meso_ is the mesopore volume. S_DFT_, V_DFT_, and L_0_(DFT) are the surface area, pore volume, and pore size obtained from DFT calculations, respectively.

**Table 2:** Mean particle size obtained from Scherrer equation.

Sample	*d*_XRD_ (nm)
NiCo_2_O_4_/Co-CX	25.5
NiFe_2_O_4_/Co-CX	19.8
CoFe_2_O_4_/Co-CX	21.8
NiCo_2_O_4_/Ni-CX	24.1
NiFe_2_O_4_/Ni-CX	21.1
CoFe_2_O_4_/Ni-CX	20.2

**Table 3:** Binding energies and chemical composition of C1s, O1s, and N1s.

Sample	C1s			O1s				N1s		
	eV	FWHM eV	Peak %	eV	Peak %	% (Mass)	% (Atomic)	eV	% (Mass)	% (Atomic)
NiCo_2_O_4_/Co-CX	284.6	1.4	70.9	529.3	36.7	28.8	32.7	399.3	0.2	0.3
	285.7		8.9	530.7	24.7			400.7		
	286.3		9.7	531.8	23.3					
	288.5		10.4	533.2	15.3					
NiCo_2_O_4_/Ni-CX	284.6	1.4	67.5	529.1	34.9	29.6	32.3	398.9	0.2	0.2
	285.6		11.0	530.7	27.8			400.4		
	286.4		10.4	531.8	22.4					
	288.5		11.1	533.2	14.9					
NiFe_2_O_4_/Co-CX	284.6	1.4	71.8	528.7	15.7	25.3	28.0	399.3	0.2	0.3
	285.7		8.8	530.1	50.4			400.7		
	286.3		9.4	531.6	24.4					
	288.5		10.1	533.1	9.5					
NiFe_2_O_4_/Ni-CX	284.6	1.4	62.5	530.1	40.0	21.9	21.6	398.7	0.6	0.7
	285.7		20.0	531.9	43.9			400.3		
	286.8		10.5	533.6	16.2					
	288.9		7.0							
CoFe_2_O_4_/Co-CX	284.6	1.6	66.9	529.7	63.9	28.6	38.5	399.4	0.4	0.7
	285.6		17.3	531.3	25.9			400.3		
	286.6		5.6	533.0	10.2					
	288.5		10.2							
CoFe_2_O_4_/Ni-CX	284.5	1.5	67.5	529.6	52.4	26.9	30.9	399.3	0.4	0.5
	285.6		12.5	531.3	35.2			400.5		
	286.4		8.3	533.1	12.5					
	288.4		11.7							

**Table 4:** XPS results collected after deconvolution of Peaks.

Sample	Fe2p_3/2_	Fe	Co2p_3/2_	Peak	Co	Ni2p_3/2_	Peak	Ni	%Fe(II)	%Fe(III)	%Fe(III)	%Fe(III)
	eV	% (Mass)	eV	%	% (Mass)	eV	%	% (Mass)	Oct	Total	Oct	Teth
NiCo_2_O_4_/Co-CX			779.2	43.0	21.6	854.3	34.4	12.1				
			780.6	57.0		855.9	65.6					
			783.9			860.9						
			788.8			863.2						
			794.4			871.9						
			796.2			873.7						
			801.6			878.7						
			804.5			881.7						
NiCo_2_O_4_/Ni-CX			779.2	43.7	18.7	854.2	32.3	11.2				
			780.6	56.3		856.0	67.7					
			784.1			860.8						
			788.8			863.5						
			794.4			871.7						
			796.2			873.5						
			802.4			878.5						
			804.8			881.5						
NiFe_2_O_4_/Co-CX	709.9	22.6				854.2	43.7	10.2	32.9	67.1	43.2	56.8
	711.5					855.6	56.3					
	713.4					860.3						
	718.7					862.5						
	723.2					871.7						
	725.1					873.2						
	727.1					877.7						
	731.8					880.6						
NiFe_2_O_4_/Ni-CX	710.0	15.6				854.3	39.3	7.8	21.9	78.1	50.2	49.8
	711.2					855.6	60.7					
	713.1					860.9						
	718.4					865.3						
	723.2					871.8						
	724.7					873.6						
	726.9					878.3						
	731.9					881.5						
CoFe_2_O_4_/Co-CX	709.8	32.7	779.6	48.7	14.2				30.7	69.3	52.7	47.3
	711.3		781.3	51.3								
	713.3		785.4									
	718.5		788.7									
	723.1		795.1									
	724.8		796.8									
	727.0		802.2									
	732.1		804.8									
CoFe_2_O_4_/Ni-CX	709.9	24.8	779.7	53.1	10.7			0.0	35.9	64.1	47.1	52.9
	711.4		781.5	46.9								
	713.3		785.4									
	718.4		788.5									
	723.1		795.1									
	724.9		796.7									
	726.7		801.7									
	732.6		804.5									

**Table 5:** Samples stoichiometries obtained from XPS.

Sample	Stoichiometry
NiCo_2_O_4_/Co-CX	$({\mathrm{Co}}_{0.86}{\mathrm{Ni}}_{0.14}$)$[{\mathrm{Co}}_{1.14}{\mathrm{Ni}}_{0.86}$]
NiCo_2_O_4_/Ni-CX	$({\mathrm{Co}}_{0.87}{\mathrm{Ni}}_{0.13}$)$[{\mathrm{Co}}_{1.13}{\mathrm{Ni}}_{0.87}$]
NiFe_2_O_4_/Co-CX	$({\mathrm{Fe}}_{0.66}{\mathrm{Ni}}_{0.34}$)${\mathrm{Fe}}_{1.24}{\mathrm{Ni}}_{0.76}$]
NiFe_2_O_4_/Ni-CX	$({\mathrm{Fe}}_{0.78}{\mathrm{Ni}}_{0.22}$)$[{\mathrm{Fe}}_{1.22}{\mathrm{Ni}}_{0.78}$]
CoFe_2_O_4_/Co-CX	$({\mathrm{Co}}_{0.34}{\mathrm{Fe}}_{0.66}$)$[{\mathrm{Fe}}_{1.34}{\mathrm{Co}}_{0.66}$]
CoFe_2_O_4_/Ni-CX	$({\mathrm{Co}}_{0.32}{\mathrm{Fe}}_{0.68}$)$\left[{\mathrm{Fe}}_{1.32}{\mathrm{Co}}_{0.68}\right]$

**Table 6:** Parameters obtained from LSV at 4000 rpm (values of n refer to K-L fitting for data at −0.8 V) and equivalent series resistance (ESR) calculated from the Nyquist plot.

Sample	E_onset_	n	ESR
	V		Ω
NiCo_2_O_4_/Co-CX	−0.06	4.0	2.74
NiFe_2_O_4_/Co-CX	−0.31	2.7	23.26
CoFe_2_O_4_/Co-CX	−0.32	2.7	15.90
NiCo_2_O_4_/Ni-CX	−0.07	3.5	6.18
NiFe_2_O_4_/Ni-CX	−0.19	2.6	10.57
CoFe_2_O_4_/Ni-CX	−0.28	2.2	22.03

## Supplementary Materials

The following are available online at https://www.mdpi.com/1996-1944/12/15/2446/s1, Figure S1: Nitrogen isotherms at −196 °C for samples: (**a**) NiFe_2_O_4_/Co-CX, **□**; NiCo_2_O_4_/Co-CX, **◇**; CoFe_2_O_4_/Co-CX, **○** and (**b**) NiFe_2_O_4_/Ni-CX, **□**; NiCo_2_O_4_/Ni-CX, **◇**; CoFe_2_O_4_/Ni-CX, **○**. Adsorption curve—open symbols; desorption curve—closed symbols. Figure S2: EDXS analysis carried out on sample NiCo_2_O_4_-CoCX.

## References

[1] Winter M., Brodd R.J. (2004). What are batteries, fuel cells and supercapacitors. Chem. Rev..

[2] Hoogers G. (2003). Fuel Cell Technology Handbook.

[3] Carrette L., Friedrich K.A., Stimming U. (2000). Fuel cells: principles, types fuels and applications. ChemPhysChem.

[4] Mahala C., Basu M. (2017). Nanosheets of NiCo_2_O_4_/NiO as efficient and stable electrocatalyst for oxygen evolution reaction. ACS Omega.

[5] Shin D., An X., Choun M., Lee J. (2016). Effect of transition metal induced pore structure on oxygen reduction reaction of electrospun fibrous carbon. Catal. Today.

[6] Wang Y.J., Zhao N., Fang B., Li H., Bi X.T., Wang H. (2015). Carbon-supported Pt-based alloy electrocatalysts for the oxygen reduction reaction in polymer electrolyte membrane fuel cells: particle size, shape, and composition manipulation and their impact to activity. Chem. Rev..

[7] Huang X., Zhao Z., Cao L., Chen Y., Zhu E., Lin Z., Li M., Yan A., Zettl A., Wang Y.M. (2015). High-performance transition metal–doped Pt_3_Ni octahedra for oxygen reduction reaction. Science.

[8] Jayasayee K., Van Veen J.R., Manivasagam T.G., Celebi S., Hensen E.J., De Bruijn F.A. (2012). Oxygen reduction reaction (ORR) activity and durability of carbon supported PtM (Co, Ni, Cu) alloys: Influence of particle size and non-noble metals. Appl. Catal. B Environ..

[9] Paulus U., Wokaun A., Scherer G., Schmidt T., Stamenkovic V., Radmilovic V., Markovic N., Ross P. (2002). Oxygen reduction on carbon-supported Pt-Ni and Pt-Co alloy catalysts. J. Phys. Chem. B.

[10] Wang B. (2005). Recent development of non-platinum catalysts for oxygen reduction reaction. J. Power Sources.

[11] Wang H., Holt C.M., Li Z., Tan X., Amirkhiz B.S., Xu Z., Olsen B.C., Stephenson T., Mitlin D. (2012). Graphene-nickel cobaltite nanocomposite asymmetrical supercapacitor with commercial level mass loading. Nano Res..

[12] Zhang G., Xia B.Y., Wang X., Lou X.W. (2014). Strongly coupled NiCo_2_O_4_-rGO hybrid nanosheets as a methanol-tolerant electrocatalyst for the oxygen reduction reaction. Adv. Mater..

[13] Chu Y.-Q., Fu Z.-W., Qin Q.-Z. (2004). Cobalt ferrite thin films as anode material for lithium ion batteries. Electrochim. Acta.

[14] Yasmin S., Cho S., Jeon S. (2018). Electrochemically reduced graphene-oxide supported bimetallic nanoparticles highly efficient for oxygen reduction reaction with excellent methanol tolerance. Appl. Surf. Sci..

[15] Gong K., Du F., Xia Z., Durstock M., Dai L. (2009). Nitrogen-doped carbon nanotube arrays with high electrocatalytic activity for oxygen reduction. Science.

[16] Pekala R., Alviso C., Kong F., Hulsey S. (1992). Aerogels derived from multifunctional organic monomers. J. Non-Cryst. Solids.

[17] Pekala R. (1989). Organic aerogels from the polycondensation of resorcinol with formaldehyde. J. Mater. Sci..

[18] EL-Deeb M.M., El Rouby W.M., Abdelwahab A., Farghali A.A. (2018). Effect of pore geometry on the electrocatalytic performance of nickel cobaltite/carbon xerogel nanocomposite for methanol oxidation. Electrochim. Acta.

[19] Zapata-Benabithe Z., Carrasco-Marín F., de Vicente J., Moreno-Castilla C. (2013). Carbon xerogel microspheres and monoliths from resorcinol–formaldehyde mixtures with varying dilution ratios: Preparation, surface characteristics, and electrochemical double-layer capacitances. Langmuir.

[20] Pérez-Cadenas A.F., Ros C.H., Morales-Torres S., Pérez-Cadenas M., Kooyman P.J., Moreno-Castilla C., Kapteijn F. (2013). Metal-doped carbon xerogels for the electro-catalytic conversion of CO_2_ to hydrocarbons. Carbon.

[21] Abdelwahab A., Castelo-Quibén J., Pérez-Cadenas M., Elmouwahidi A., Maldonado-Hódar F.J., Carrasco-Marín F., Pérez-Cadenas A.F. (2017). Cobalt-Doped Carbon Gels as Electro-Catalysts for the Reduction of CO_2_ to Hydrocarbons. Catalysts.

[22] Moreno-Castilla C., Maldonado-Hódar F. (2005). Carbon aerogels for catalysis applications: An overview. Carbon.

[23] Abdelwahab A., Castelo-Quibén J., Vivo-Vilches J.F., Pérez-Cadenas M., Maldonado-Hódar F.J., Carrasco-Marín F., Pérez-Cadenas A.F. (2018). Electrodes Based on Carbon Aerogels Partially Graphitized by Doping with Transition Metals for Oxygen Reduction Reaction. Nanomaterials.

[24] Maldonado-Hódar F., Moreno-Castilla C., Pérez-Cadenas A. (2004). Surface morphology, metal dispersion, and pore texture of transition metal-doped monolithic carbon aerogels and steam-activated derivatives. Microporous Mesoporous Mater..

[25] Job N., Pirard R., Marien J., Pirard J.-P. (2004). Synthesis of transition metal-doped carbon xerogels by solubilization of metal salts in resorcinol–formaldehyde aqueous solution. Carbon.

[26] Moreno-Castilla C., Maldonado-Hódar F., Pérez-Cadenas A. (2003). Physicochemical surface properties of Fe, Co, Ni, and Cu-doped monolithic organic aerogels. Langmuir.

[27] Abdelwahab A., Castelo-Quibén J., Pérez-Cadenas M., Maldonado-Hódar F.J., Carrasco-Marín F., Pérez-Cadenas A.F. (2018). Insight of the effect of graphitic cluster in the performance of carbon aerogels doped with nickel as electrodes for supercapacitors. Carbon.

[28] Chmiola J., Yushin G., Dash R., Gogotsi Y. (2006). Effect of pore size and surface area of carbide derived carbons on specific capacitance. J. Power Sources.

[29] Gryglewicz G., Machnikowski J., Lorenc-Grabowska E., Lota G., Frackowiak E. (2005). Effect of pore size distribution of coal-based activated carbons on double layer capacitance. Electrochim. Acta.

[30] de Lima Alves T.M., Amorim B.F., Torres M.A.M., Bezerra C.G., de Medeiros S.N., Gastelois P.L., Outon L.E.F., de Almeida Macedo W.A. (2017). Wasp-waisted behavior in magnetic hysteresis curves of CoFe_2_O_4_ nanopowder at a low temperature: experimental evidence and theoretical approach. RSC Adv..

[31] Yang Y., Zeng D., Yang S., Gu L., Liu B., Hao S. (2019). Nickel cobaltite nanosheets coated on metal-organic framework-derived mesoporous carbon nanofibers for high-performance pseudocapacitors. J. Colloid Interface Sci..

[32] Jokar E., Shahrokhian S. (2015). Synthesis and characterization of NiCo_2_O_4_ nanorods for preparation of supercapacitor electrodes. J. Solid State Electrochem..

