# Supplementary material for: Carbon Xerogels Hydrothermally Doped with Bimetal Oxides for Oxygen Reduction Reaction
Source: Materials (Basel). 2019 Jul 31;12(15):2446. doi: 10.3390/ma12152446 (PMC6696479; doi:10.3390/ma12152446)
Supplement: Supplementary file 1 [file materials-12-02446-s001.pdf]

materials

Supporting Information

Carbon Xerogels Hydrothermally Doped with Bimetal Oxides for Oxygen Reduction Reaction

Abdalla Abdelwahab [1,*], Francisco Carrasco-Marín [2] and Agustín F. Pérez-Cadenas [2,*]

1 Materials Science and Nanotechnology Department, Faculty of Postgraduate Studies for Advanced Sciences, Beni-Suef University, Beni-Suef 62511, Egypt
2 Carbon Materials Research Group, Department of Inorganic Chemistry, Faculty of Sciences, University of Granada, Campus Fuentenueva s/n, ES18071 Granada, Spain
* Correspondence: aabdelwahab@psas.bsu.edu.eg (A.A.); afperez@ugr.es (A.F.P.-C.)

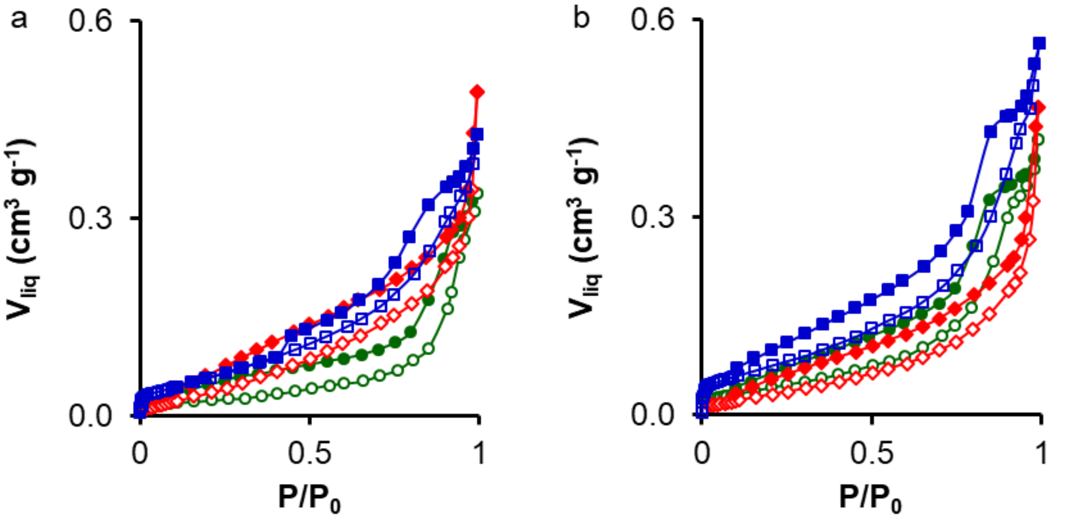

(**a**) (**b**)

Figure S1. Nitrogen isotherms at −196 °C for samples: (**a**) $NiFe_2O_4$/Co-CX, □; $NiCo_2O_4$/Co-CX, ◇; $CoFe_2O_4$/Co-CX, ○ and (**b**) $NiFe_2O_4$/Ni-CX, □; $NiCo_2O_4$/Ni-CX, ◇; $CoFe_2O_4$/Ni-CX, ○. Adsorption curve—open symbols; desorption curve—closed symbols.

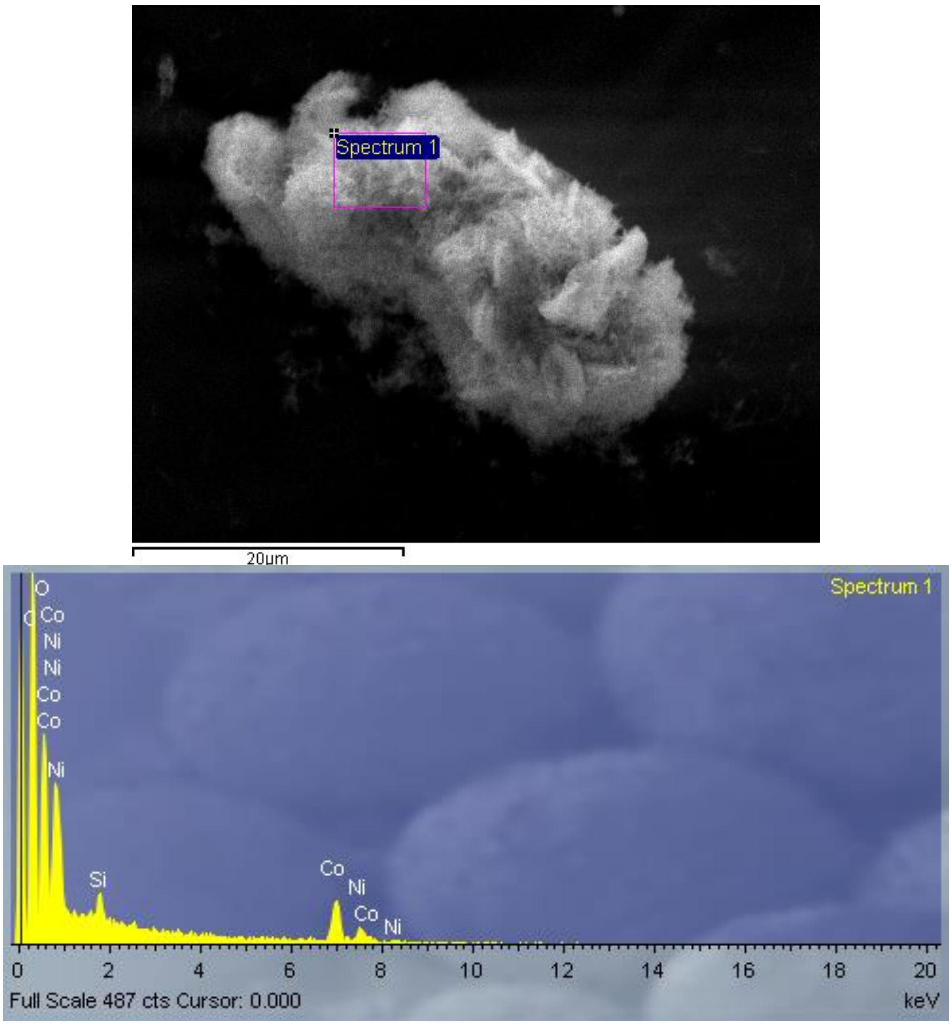

Figure S2. EDXS analysis carried out on sample $NiCo_2O_4$-CoCX.

© 2019 by the authors. Submitted for possible open access publication under the terms and conditions of the Creative Commons Attribution (CC BY) license (http://creativecommons.org/licenses/by/4.0/).
